# Epinephrine soaked tampons induced transient acute dilated cardiomyopathy during FESS procedure

**DOI:** 10.1186/s12872-020-01706-8

**Published:** 2020-10-16

**Authors:** Sari Naddaf, Scott Ehrenberg, Rony Hakim, Muhamad Mahamid, Yoav Turgeman, Ofir Koren

**Affiliations:** 1 Bruce Rappaport Faculty of Medicine, Technion-Israel Institute of Technology, Haifa, Israel; 2 Department of Anaesthesia, Emek Medical Center, Afula, Israel; 3 Heart Institute, Emek Medical Center, Afula, Israel

**Keywords:** Epinephrine, Dilated cardiomyopathy, Heart failure, FESS, Surgery

## Abstract

**Background:**

Epinephrine, in all modes of use, may pose a wide range of cardiotoxic events, ranging from sinus tachycardia to heart failure, life threatening arrhythmias, and even death. Because of daily and extensive use of epinephrine, these unusual and rare events tend to be forgotten by physicians. We present a case of dilated cardiomyopathy that developed following routine use of epinephrine-impregnated tampons during function endoscopic sinus (FESS) surgery.

**Case presentation:**

A healthy, 24-year-old man with no family history of heart disease has undergone elective surgery under general anesthesia to repair the paranasal sinuses using endoscopic approach. During surgery, soon after being treated with 1: 1000 diluted epinephrine-soaked tampons, an hypertensive crisis was noticed followed by pulseless electrical activity. An extensive examination led to the diagnosis of non-ischemic dilated cardiomyopathy. After several days of heart failure medical therapy, complete resolution of all structural and functional changes was achieved.

**Conclusion:**

In our case, we present an unusual and rare event of acute dilated cardiomyopathy following the use of epinephrine-soaked tampons during elective FESS surgery. A prompt response was observed after several days of heart failure treatment. Awareness of the epinephrine cardiotoxic potential even in the form of soaked tampons is essential for proper diagnosis and prompt treatment.

## Background

Dilated cardiomyopathy (DCM) is recognized by the dilation of the right, left, or both ventricles in the absence of abnormal loading conditions as hypertension and valve disease, or significant coronary artery disease [1–5].

The etiology of DCM is extremely heterogeneous sometimes classified based on known genetic mutation. Among the non-genetics cause are different etiologies, including myocarditis, exposure to drugs as cocaine, certain toxins as alcohol or allergens; complication of pregnancy, systemic endocrine or autoimmune diseases, and infection as HIV [6].

One of the rarest etiologies regarding toxin and metabolic-related cause of DCM is catecholamines, and its various derivates, which has been reported mainly in case reports or short series review [7–13].

Pheochromocytoma, as a rare neuroendocrine catecholamine-producing tumor, has also been described as an etiology of reversible DCM and was found in ~ 39% of pheochromocytoma-related cardiomyopathies [14]. Paul et al. proposed that catecholamine-induced vasoconstriction, ﻿a direct toxic effect of the by-products of catecholamine degradation and direct receptor-mediated mechanisms, contribute to cardiomyopathy in subjects with pheochromocytoma [15].

## Case description

**Fig. 1:**
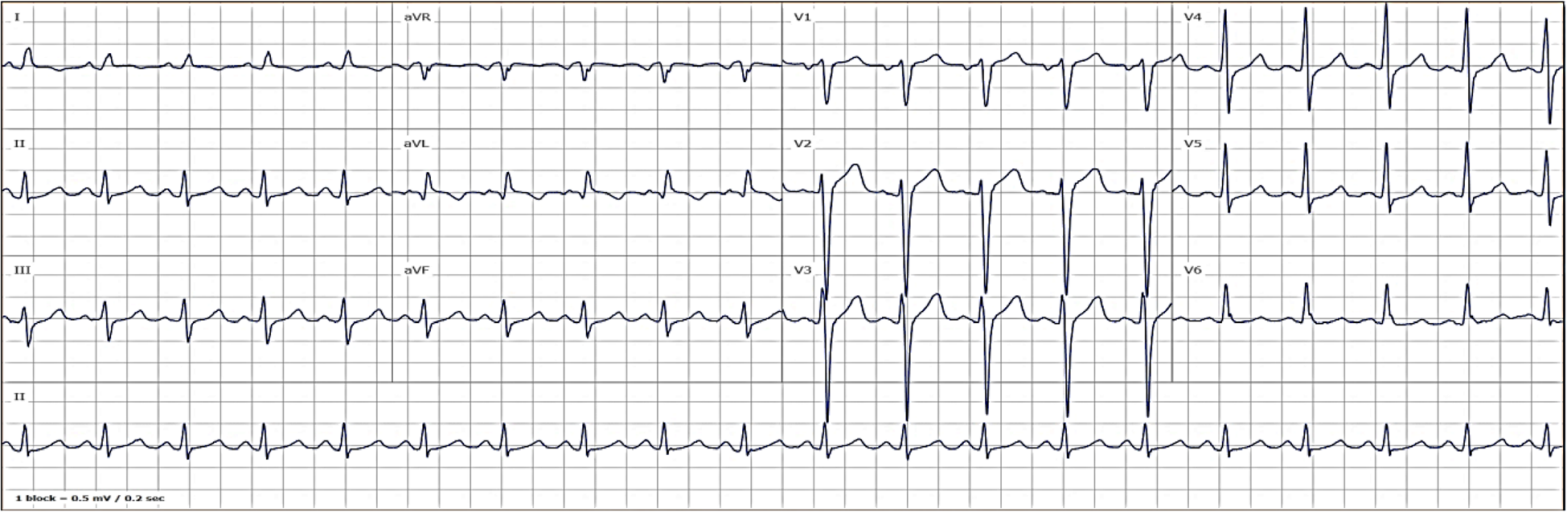
ECG at admission indicate sinus tachycardia, prolonged QTc (QTc = 486 ms), Inverted T waves in leads I and aVL, and no signs of acute Ischemic changes

**Fig. 2:**
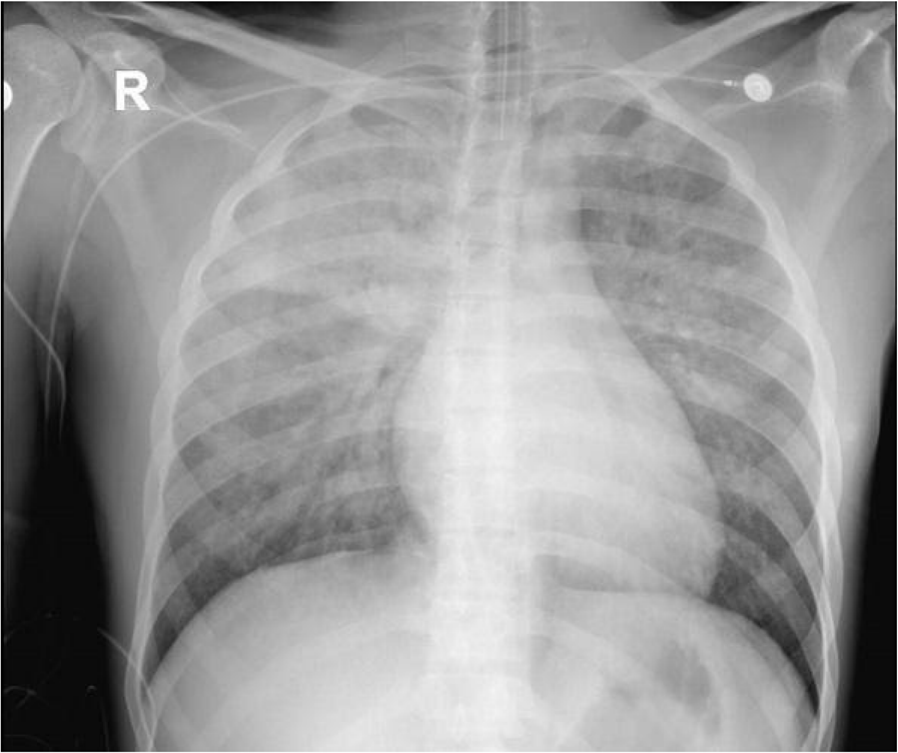
Chest X-ray at admission indicate bilateral pulmonary edema and normal heart silhouette

A 24-year old male with chronic rhinosinusitis was admitted for an elective Functional endoscopic sinus surgery (FESS) procedure under general anesthesia. During the operation, the surgeon applied several tampons soaked in 1:1000 dilution epinephrine to the nasal mucosa. Ninety minutes into the surgery, unexpectedly, blood pressure rose to 210/130 mmHg followed by pulseless electrical activity. CPR was initiated, with the administration of 2 mg of IV epinephrine in consecutive doses, leading to the return of spontaneous circulation. ECG showed sinus tachycardia and a prolonged QTc interval of 486 ms, Inverted T waves in leads I and aVL, and no signs of acute ischemic changes (Fig. 1). A chest x-ray demonstrated pulmonary edema and a borderline enlarged cardiac silhouette (Fig. 2).

**Fig. 3:**
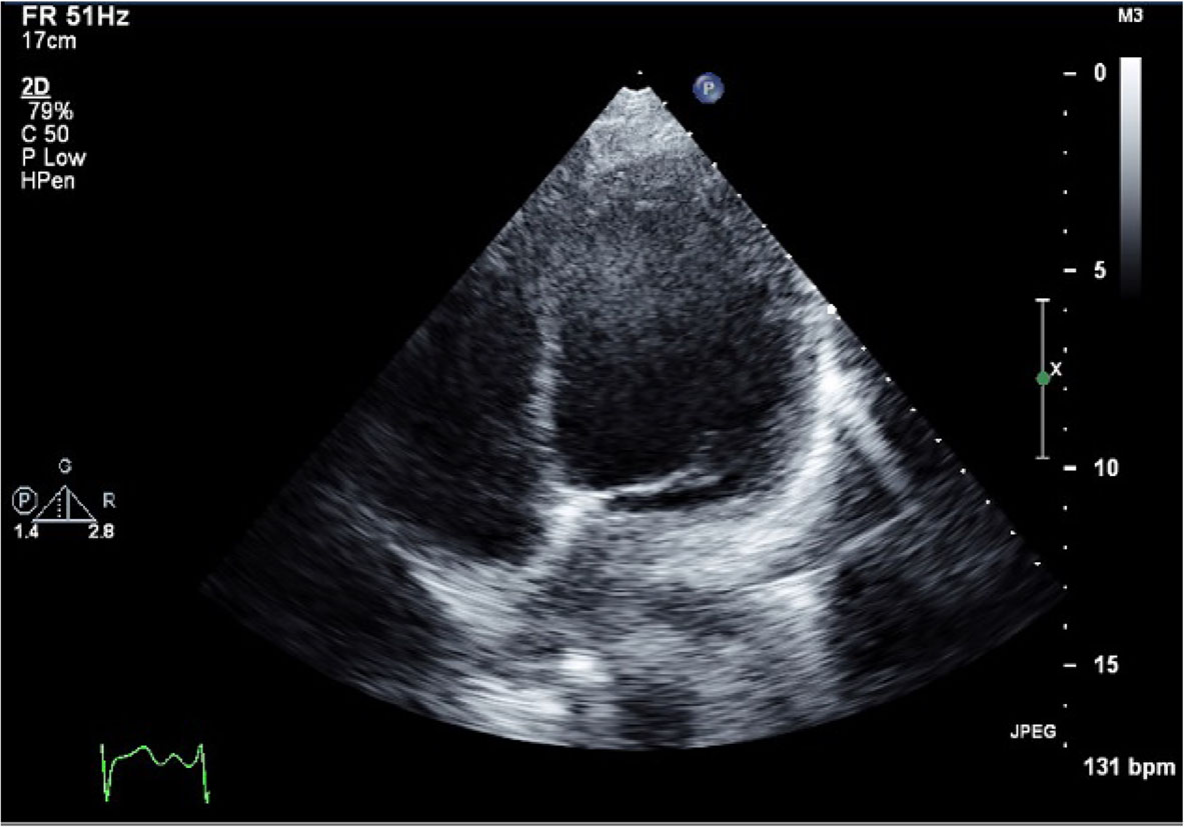
Transthoracic Echocardiography at admission demonstrates a dilated left ventricle dimension. Apical 4 Chambers view at the end of the diastole

**Fig. 4:**
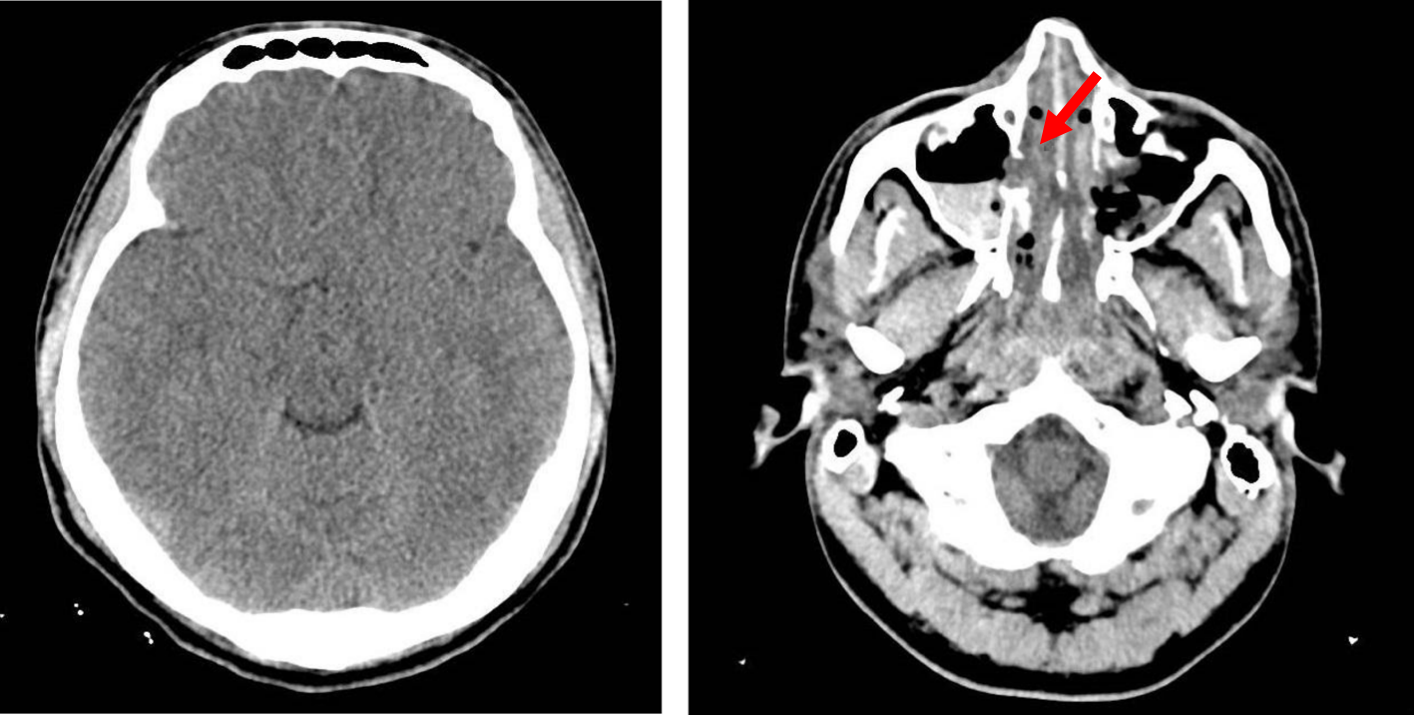
Head CT at admission shown Global Edema (Left-sided) and multiple Maxillary Sinuses fractures (Right-sided red arrow)

The patient was placed on mechanical ventilation. Transthoracic echocardiogram showed a dilated left ventricle with an increased end-diastolic dimension (124% of normal value) with a mild reduction in LV mass, a severe reduction in systolic function, apical akinesis, and hyperdynamic base. The estimated systolic left ventricular ejection fraction was 30% (Fig. 3). Cardiac troponin and CPK were elevated. NT or NT pro-BNP were not taken. A head CT was performed and demonstrated mild global cerebral edema, with multiple maxillary sinus fractures (Fig. 4).

Medical information gathered from family members indicated no family history of heart diseases. The patient did not smoke, use illegal drugs nor consumed alcohol on daily basis. The patient was diagnosed with dilated cardiomyopathy and admitted to the intensive cardiac care unit. Upon arrival, upload titration of ACE inhibitors, β-blockers, and diuretics initiated. The following ECG strips indicate normal sinus rhythm, QTc interval of 420 ms, and normal T wave in lateral leads. We decided not to perform a diagnostic coronary catheterization since the patient's risk profile for ischemic cardiomyopathy was low. Objectively, there were no ischemic changes on ECGs strips and no regional wall motional abnormalities were seen on TTE upon arrival.

**Fig. 5:**
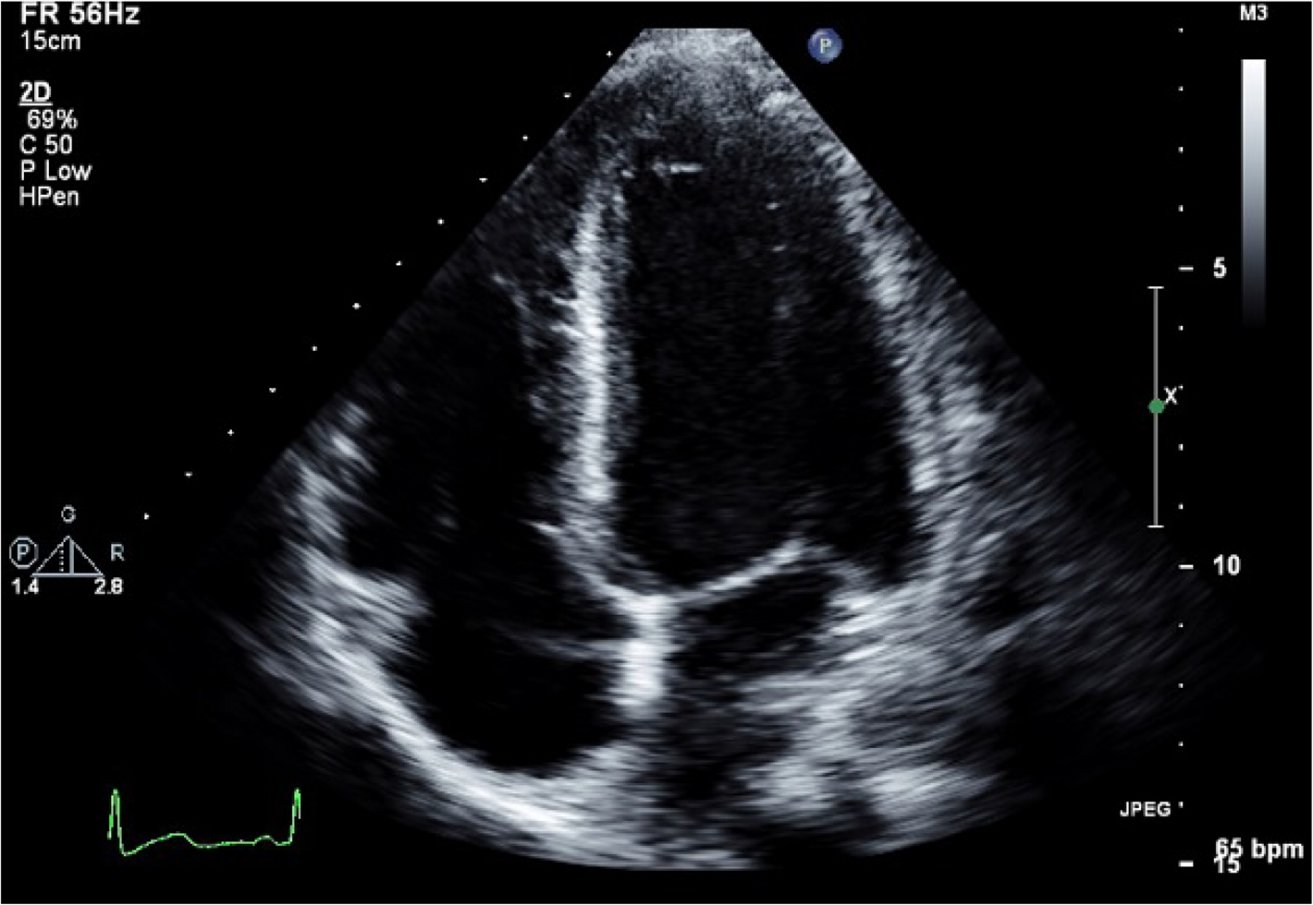
Transthoracic Echocardiography at discharge demonstrates a normal-sized left ventricle dimension with normal LV mass. Apical 4 Chambers view at the end of the diastole

Three days later, a second echocardiogram was performed which showed a normal-sized left ventricle, with preserved systolic function (Fig. 5).

The patient was discharged in full functional capacity. An ambulatory cardiac MRI performed two weeks after discharge revealed mildly dilated LV cavity, good global systolic function, and no signs of late gadolinium enhancement or edema.

## Discussion

The relationship between exogenous catecholamines and dilated cardiomyopathy is not fully understood. Some propose that elevated catecholamine levels decrease myocardial viability via cyclic -AMP mediated Ca + overload [16, 17].

While reductions in ejection fraction and left ventricular dysfunction was found to be reversible, mild histologic changes were found in dog studies indicate chronic perivascular fibrosis which seems to be irreversible [18].

Since activation of alpha or adrenergic receptors has been shown to induce stress-induced cardiomyopathy, it has been shown in studies that these cardiac effects can be attenuated by pretreatment with the use of alpha and beta blockers [19–23]. In addition, increasing levels of estrogen have shown partial attenuation of these cardiac changes [24, 25].

Dilated cardiomyopathy is usually characterized by long-standing processes or conditions, its appearance in the acute setting is not common and a detailed evaluation of acute onset dilated cardiomyopathy (ADCM) typically does not elucidate a specific etiology in most cases [26].

## Conclusion

We report a rare case of a transient acute non-ischemic transient dilated cardiomyopathy following exposure to sstandard diluted dose epinephrine soaked-tampons during ENT procedure. Prompt heart failure treatment resulted in complete resolution. Our report provides supporting evidence of the cardiotoxic devastating potential effect of epinephrine and its role in acute dilated cardiomyopathy. We hope that this paper will raise awareness among physicians and surgeons to the relevance of cardiotoxic effect of epinephrine, especially in the form of soaked tampons.

## Data Availability

The datasets used and/or analyzed during the current study are available from the corresponding author on reasonable request.

## Abbreviations

FESS: Functional endoscopic sinus surgery
ENT: Ear, nose and throat
DCM: Dilated cardiomyopathy
ECG: Electrocardiography
TTE: Transthoracic echocardiography
CT: Computerized tomography
MRI: Magnetic resonance imaging
PEA: Pulseless electrical activity
CPR: Cardiopulmonary resuscitation
ACEI: Angiotensin-converting enzyme inhibitor
BB: Beta-blocker
